# Parametric Modeling of Human Gradient Walking for Predicting Minimum Energy Expenditure

**DOI:** 10.1155/2015/407156

**Published:** 2015-08-31

**Authors:** Gerard Saborit, Adrià Casinos

**Affiliations:** Departament de Biologia Animal, Universitat de Barcelona, 08028 Barcelona, Spain

## Abstract

A mathematical model to predict the optimum gradient for a minimum energetic cost is proposed, based on previous results that showed a minimum energetic cost when gradient is −10%. The model focuses on the variation in mechanical energy during gradient walking. It is shown that kinetic energy plays a marginal role in low speed gradient walking. Therefore, the model considers only potential energy. A mathematical parameter that depends on step length was introduced, showing that the optimal gradient is a function of that parameter. Consequently, the optimal negative gradient depends on the individual step length. The model explains why recent results do not suggest a single optimal gradient but rather a range around −10%.

## 1. Introduction

Human walking requires energy for a variety of reasons. For instance, in level walking, alternate stages of braking and acceleration exist. Although there is a pendulum-like transfer between potential and kinetic energy of the body center of mass, this is only an energy-saving system. Since the transfer is not complete, additional energy must be incorporated into the system in each step (Cavagna et al. [1, 2]).

In gradient walking the situation changes depending on whether walking up- or downhill. In the former case (positive gradient) positive work is needed to provide gravitational potential energy. In downhill walking, the lost potential energy is absorbed by muscles compelled to stretch. Cavagna [3] showed that the lost energy is transformed into heat through negative or braking work. Direct experiments measuring oxygen uptake in subjects walking on different gradients showed that the minimum energetic cost is not accomplished on level groundbut on a negative gradient of about −10% (Margaria [4]). Further studies (Minetti et al. [5]) demonstrated that minimum energetic cost does not depend on speed and that the optimal path within a positive gradient, considering the vertical cost of transportation, is not always the straight one (Minetti [6]). Further studies (Kamon [7]) showed that oxygen uptake during descent can be about 30% of that required during ascent. Therefore we can deduce that the process of muscular braking, which involves negative work, is energetically different from positive work due to different efficiency factors [8–11]. A complete mechanical analysis must include both kinetic and potential energies but we can calculate each contribution to determine whether one of these (kinetic or potential) is more dominant or whether both energies contribute equally to the whole energetic cost.

Since walking implies low and rather constant velocity, kinetic energy does not vary greatly during the different walking phases. Supposing standard walking at a speed of 1.25 m·s^−1^, the total kinetic energy involved in the movement is 0.78 J per unit mass. This energy is not supplied at every step since people do not come to a complete standstill between steps. During walking the center of mass moves at almost constant speed. Gottschall and Kram [12] quantified the variation in velocity of the center of mass during different step phases and for different gradients. This variation is about 0.09 m·s^−1^per step for level walking with a maximum of 0.18 m·s^−1^ per step in some downhill walking situations. During each step one brakes and accelerates about 0.09 m·s^−1^, leading to a small variation in speed (from 1.20 m·s^−1^ to 1.30 m·s^−1^). Calculation of the energy per unit mass taking this speed variation into account shows that the kinetic energy per unit mass needed is about 0.12 J·kg^−1^ per step for level walking and up to 0.20 J·kg^−1^per step for high negative gradients.

For potential energy, the vertical oscillation of the center of mass varies from 8 to 10 cm, depending on step length. This means a potential energy oscillation per unit mass from 0.78 J·kg^−1^ per step up to 0.98 J·kg^−1^ per step. It is clear that kinetic energy plays a lesser role in walking at low speeds, being from 5 to almost 10 times smaller than potential energy depending on a number of variables. Another factor to take into account is that the transfer of energy from one walking phase to another usually transforms the excess potential energy, achieved during the single support phase, to kinetic energy for the body. The kinetic energy of the center of mass is almost constant and the main loss of kinetic energy is due to the contact between the still feet on the ground and braking work to avoid acceleration. In the next step phase, the muscles perform positive work to raise the center of mass, thus gaining potential energy again. This is another reason for focusing the analysis on potential energy: the energy transfer involves transforming potential energy into kinetic energy in such a way that the calculation done above for kinetic energy could be overestimated. The significant effect of gravity on walking has been evaluated in previous studies [5, 13] and the incomplete energetic transfer between potential and kinetic energy during walking has been widely discussed [14–17]. As shown by Heglund and Schepens [18], this energy transfer also varies depending on age, with less recovery in children than in adults.

For the reasons stated above, this work focuses on the variation in potential energy during the walking process, as a simple and first approximation analysis. Further corrections such as kinetic energy components could be introduced if the model's predictions are not sufficiently accurate. The main objective of the model is to prove that the vertical oscillation of the center of mass is ultimately responsible for the minimum energy spent at low negative gradient and that only with potential energy analysis will the model fit previous experimental results.

## 2. Model

In human walking there are basically two stages. In the first stage, the feet are simultaneously on the ground (double support) and the center of mass is at its lowest point, at a distance *y*_min_ from the ground. In the second stage, one foot is on the ground (single support), with the corresponding leg straight. The center of mass is at its highest position, at a maximum distance from the ground (*y*_max_).

Consider now a human with leg length *l*. Usually the step length tends to be smaller than the leg length. Thus it can be modeled as *l*/*k*, with the variable *k* being an arbitrary parameter that differs for each individual, within a range.

**Figure 1:**
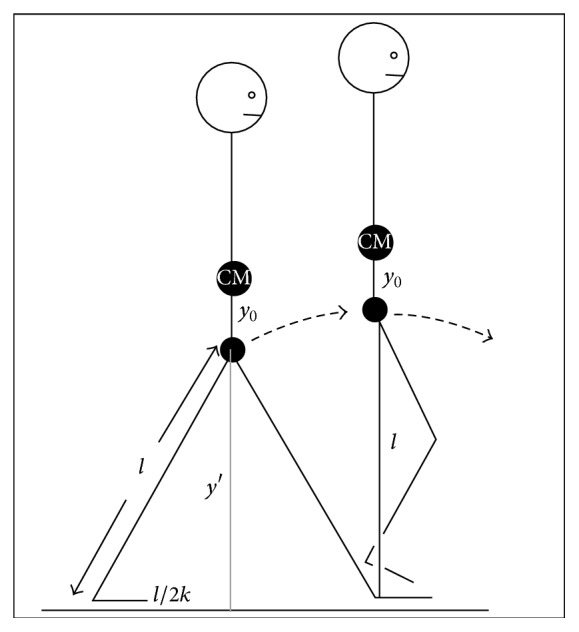
Height variation of the human center of mass during successive step phases. Double support phase (left) and single support phase (right) are shown. Extracted and modified from Alexander [19].

As shown in Figure 1, the maximum height of the center of mass occurs during the single support phase and can be defined as follows:

(1) $$\begin{array}{c} y_{max}=y_{0}+l, \end{array}$$

where *y*_0_ is the vertical distance from the center of mass to the acetabular joint and *l* is the distance from the acetabular joint to the ground. That means *l* is the leg length.

On the other hand, the minimum height of the center of mass occurs during the double support phase and can be defined as

(2) $$\begin{array}{c} y_{min}=y_{0}+y^{\prime }, \end{array}$$

where *y*′ is the distance from the acetabular joint to the ground and *y*_0_ is, again, the distance from this joint to the center of mass. Hence the distance between the acetabular joint and the ground can be defined as a cathetus of a right-angled triangle, the half-step ground distance (*l*/2*k*) as the other cathetus, and the leg length (*l*) as the hypotenuse.

From Figure 1, the right triangle is defined using the Pythagorean theorem:

(3) $$\begin{array}{c} l^{2}={y^{\prime }}^{2}+\frac{l^{2}}{4k^{2}}. \end{array}$$

*y*′can be found from (3):

(4) $$\begin{array}{c} y^{\prime }=\sqrt{l^{2}-\frac{l^{2}}{4k^{2}}}=l\sqrt{1-\frac{1}{4k^{2}}}. \end{array}$$

Replacing the *y*′ in (2) we find

(5) $$\begin{array}{c} y_{min}=y_{0}+l\sqrt{1-\frac{1}{4k^{2}}}. \end{array}$$

The total oscillation of the center of mass Δ*y* can be defined as

(6) $$\begin{array}{c} \Delta y=y_{max}-y_{min}. \end{array}$$

Using the values found in (1) and (5),

(7) $$\begin{array}{c} \Delta y=y_{0}+l-y_{0}-l\sqrt{1-\frac{1}{4k^{2}}} \end{array}$$

and subsequently

(8) $$\begin{array}{c} \Delta y=l\left(1-\sqrt{1-\frac{1}{4k^{2}}}\right). \end{array}$$

By definition, the variation in potential energy Δ*U* of a body that suffers a change in height is

(9) $$\begin{array}{c} \Delta U=mg\Delta y. \end{array}$$

Replacing the value found in (8) in (9),

(10) $$\begin{array}{c} \Delta U_{osc}=mgl\left(1-\sqrt{1-\frac{1}{4k^{2}}}\right). \end{array}$$

Equation (10) is the total variation in potential energy of the center of mass during the oscillation that occurs at each step. It is termed Δ*U*_osc_, to differentiate it from any other potential energy variation.

Let us consider now the variation in potential energy due to a gradient. Conditions differ depending upon the sign of the gradient, but for mathematical simplicity a positive gradient will be considered. It should be noted that during gradient walking there is an adaptation of the step phases and they may not necessarily occur at the same point as in level walking. For instance, in downhill walking the rising of the center of mass is done faster than in level walking, and the later decline is done slower. In any case the height variation of the center of mass is not different than in level walking. So in terms of energy it is indifferent whether the elevation of the center of mass happens sooner or later, as at each step very similar variations of height occur. For simplicity of this first approximation model we suppose that the different timing of the walking phases between gradient and level walking does not affect significantly the energetic calculation.

**Figure 2:**
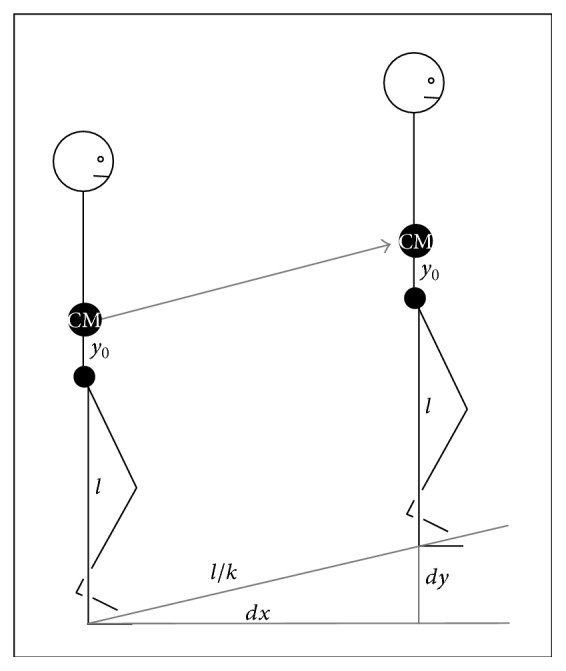
Gradient that a human overcomes in one step during uphill walking. Extracted and modified from Alexander [19].

Take the height attained (*dy*) in a gradient with the step length (*l*/*k*). These two distances and the horizontal projection of the step length (*dx*) define a right triangle (Figure 2).

By definition, the gradient (*i*) is the height variation per unit of displacement:

(11) $$\begin{array}{c} i=\frac{dy}{dx}. \end{array}$$

Therefore, the height variation in terms of the gradient is

(12) $$\begin{array}{c} dy=idx. \end{array}$$

The sides of the right triangle are defined using the Pythagorean theorem:

(13) $$\begin{array}{c} \frac{l^{2}}{k^{2}}=dy^{2}+dx^{2}. \end{array}$$

Replacing *dy* by its value found in (12),

(14) $$\begin{array}{c} \frac{l^{2}}{k^{2}}={\left(idx\right)}^{2}+dx^{2}=dx^{2}\left(1+i^{2}\right). \end{array}$$

Solving (14) for *dx* we find

(15) $$\begin{array}{c} dx^{2}=\frac{l^{2}}{k^{2}\left(1+i^{2}\right)} \end{array}$$

and finally

(16) $$\begin{array}{c} dx=\frac{l}{k}\sqrt{\frac{1}{1+i^{2}}}. \end{array}$$

The variation in the potential energy is defined in (9). Using (12) again, the variation in potential energy due to a certain gradient for one step (Δ*U*_grad_) is

(17) $$\begin{array}{c} ∆U_{grad}=mgdy=mgidx \end{array}$$

and using the value of *dx* found in (16),

(18) $$\begin{array}{c} \Delta U_{grad}=mgi\frac{l}{k}\sqrt{\frac{1}{1+i^{2}}}. \end{array}$$

Equation (18) defines the energy that must be supplied in one step to overcome gradient *i*. If the gradient is negative, that amount of energy would be supplied to the body instead, and, consequently, it would accelerate unless the subject brakes. According to previous results [1, 7], braking work requires four to five times less energy than positive work. In other words, to absorb and brake 100 J of potential energy, transformed to kinetic energy during downhill walking, the body does about 20 J of negative work. The negative work required to brake is proportional to the energy previously transferred in the form of kinetic energy.

The total potential energy contribution to the whole energetic cost is the sum of two main factors: one for the oscillation of the center of mass due to the walking process and the other to overcome a given variable gradient, if any.

Thus, the total potential energy contribution (*U*_tot_) can be written as the sum of these two factors:

(19) $$\begin{array}{c} U_{tot}=\Delta U_{grad}+\Delta U_{osc}. \end{array}$$

It must be taken into account that if the total energy is negative, that is, the body receives energy from a negative gradient, it must brake to avoid acceleration. As mentioned previously, the negative work is about five times more efficient than positive work. To reflect this, an auxiliary function *ε* is defined, which includes the cost of negative work, if necessary. If the total amount of energy is positive, the body must supply that energy, as it must overcome a certain gradient. If it is negative an excess of potential energy is received due to the loss of height. So braking work five times smaller than the excess of potential energy received must be performed [7]. As absolute work is positive, the factor introduced to reflect the difference in efficiency is −5, to ensure that *ε* is positive for negative gradients:

(20) ε=Utot if  Utot>0,ε=Utot−5 if  Utot<0.

**Figure 3:**
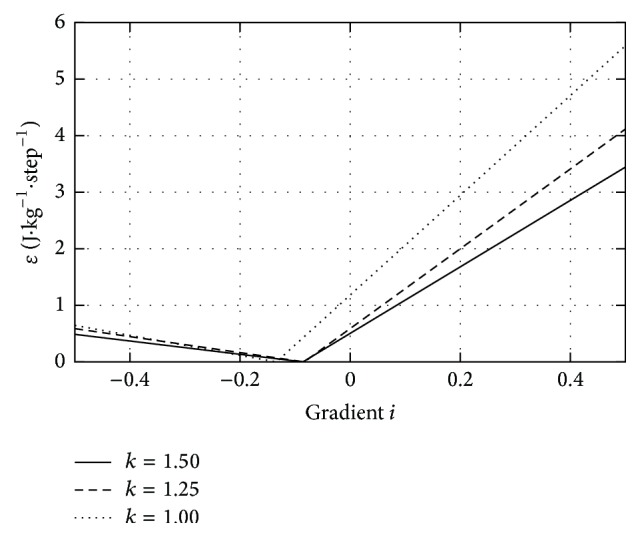
Total potential energy contribution to the energy expenditure as a function of gradient *i*. Multiple plots were constructed assuming *k* = 1, *k* = 1.25, and *k* = 1.5. All lines were constructed assuming *g* = 9.81 m·s^−2^.

Plotting *ε* as a function of the gradient (*i*) gives Figure 3, with very similar behavior to the experimental results found in Margaria [4] and Minetti et al. [5] for gradient walking and even Minetti's research [20] on gradient running.

Figure 3 faithfully reproduces experimental data found in previous oxygen consumption experiments. The fact that we can reproduce this behavior with such a simple model confirms our hypothesis: as a first approximation kinetic energy variation is not relevant for low speed walking and the timing fluctuations between stages in gradient and level walking are insignificant in energetic terms. In addition to reproducing the pattern of oxygen consumption, our model predicts the optimal gradient and links it to step length, as this minimum varies with the value of the *k* parameter, which depends on step length. To find an analytic expression of the optimum gradient in terms of *k*, that is, in terms of step length, Δ*U*_grad_ and Δ*U*_osc_ must be the same since at the minimum energetic cost they are equal but with opposite sign. This means their sum is zero:

(21) $$\begin{array}{c} \Delta U_{grad}+\Delta U_{tot}=0. \end{array}$$

Taking the values found in (18) and (10) for Δ*U*_grad_ and Δ*U*_osc_, respectively, each term is replaced:

(22) $$\begin{array}{c} mgl\frac{i}{k}\sqrt{\frac{1}{1+i^{2}}}+mgl\left(1-\sqrt{1-\frac{1}{4k^{2}}}\right)=0. \end{array}$$

It should be noted that for low gradients (*i* ≈ 0) the first square root term is close to 1. For mathematical simplicity this root can be removed from the equation, without introducing any significant error, resulting in

(23) $$\begin{array}{c} mgl\frac{i}{k}+mgl\left(1-\sqrt{1-\frac{1}{4k^{2}}}\right)=0. \end{array}$$

Developing and taking out the common factor *mgl*,

(24) $$\begin{array}{c} \frac{i}{k}+1-\sqrt{1-\frac{1}{4k^{2}}}=0. \end{array}$$

Solving the equation for *i* gives

(25) $$\begin{array}{c} \frac{i}{k}=\sqrt{1-\frac{1}{4k^{2}}}-1 \end{array}$$

and finally

(26) $$\begin{array}{c} i=k\sqrt{1-\frac{1}{4k^{2}}}-k. \end{array}$$

**Figure 4:**
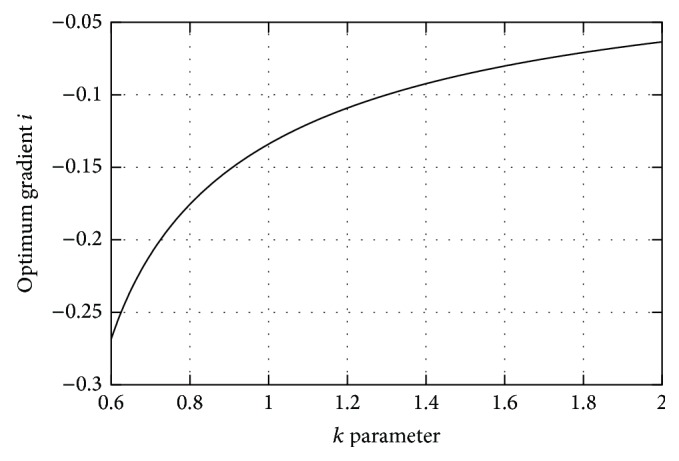
Optimum gradient as a function of the *k* parameter.

Figure 4 shows the plot of *i*(*k*) function found in (26). As defined above, the *k* parameter is a function of step length, so it can be shown that there is an optimum gradient, around −10%, for a given step length but that, depending on that step length, the optimum gradient can vary somewhat. For large step lengths (*k* ~ 1.10), the optimum gradient tends to be around −12%, and for short step lengths (*k* ~ 1.5), the optimum gradient tends to be lower, around −8%. Alexander [21] inferred that the range of comfortable variation in step length is *k* = 1.35 ± 0.20. So the optimal gradient of a given subject can usually be expected to be around −10%.

## 3. Conclusion

The model, which is based on analysis of the variation in potential energy during walking, fits the experimental results on minimum energy expenditure obtained in previous studies and links this minimum to the step length of the subject. The hypothesis that kinetic energy plays a small role at low speeds and is not needed in a first approximation of the mechanical analysis of gradient walking appears to be correct.

The model proves mathematically that the minimum energy expenditure is due to potential energy exchange and is related to the subject's step length. The reason for this is that in every step the center of mass is raised and the body must supply some potential energy, but with a low negative gradient, this energy can be supplied instead by the loss of potential energy. In these circumstances the body saves energy and can move in a more efficient way, requiring less oxygen uptake.

Our work demonstrates that the negative gradient that minimizes energy expenditure depends on the *k* parameter. For long steps the potential energy involved is higher since the variation in height of the center of mass is bigger, and for short steps the variation in height of the center of mass is smaller. Therefore each step length has its own negative gradient of minimum energy expenditure. This suggests new strategies for minimizing energy expenditure during gradient walking. A solution for minimizing the energy spent in either downhill or uphill walking would be to adjust the step length for a given variable gradient to ensure that the subject remains on the optimal gradient range.

Common walking strategies for minimizing the energetic cost of movement usually involve either changing step length or stride frequency [22, 23]. Decreasing the stride frequency leads to variation in the walking velocity, similarly to step length decrease and therefore a decrease in kinetic energy and potential energy expenditure too, since the center of mass must be raised fewer times in a given period. Step length variation can be considered a valid strategy because people can slightly modify step length within a range in a comfortable fashion. In our model the possibility of step length modification is reflected in the range of variation of the *k* parameter (1.35 ± 0.20). This means that, assuming a leg length of 90 cm, people can comfortably use a step length range of 60–80 cm. Longer or shorter steps outside this range are possible, but it would be uncomfortable to maintain this strategy over long periods. Given the range of variation mentioned above, it can be seen from Figure 4 that the optimum gradient range can easily extend from −8% to −12% by adjusting the step length to keep *k* between 1.10 and 1.50. From Figure 4 it can be seen that for gradients beyond −13% the optimal step length (*k* = 1) is too wide to be considered as a valid strategy. In such circumstances other strategies could be used, such as lowering the stride frequency and step length, but with the drawback of reducing the walking velocity. Leroux et al. [22] suggested that step length is slightly reduced as negative gradients increase beyond the comfortable range. This fits with the stated strategy of reducing the walking velocity, or keeping the same velocity by adjusting the stride frequency while reducing step length, as the body needs to absorb more energy from the negative gradient to avoid acceleration, and the more negative the gradient is, the more braking work needs to be done.

In conclusion, this work presents a parametric model based on an analysis of the variation in potential energy during gradient walking, which explains the energetic mechanism behind the minimum energy spent experimentally in many previous studies, and links this optimum gradient with step length.
